# Juvenile diabetes and systemic sclerosis: just a coincidence?

**DOI:** 10.1186/s12969-022-00741-3

**Published:** 2022-09-11

**Authors:** Greta Mastrangelo, Alessandra Meneghel, Giorgia Martini, Carlo Moretti, Francesco Zulian

**Affiliations:** 1 Division of Rheumatology, Department of Paediatrics, The Hospital for Sick Children, 555 University Ave, Toronto, ON M5G 1X8 Canada; 2 Pediatric Rheumatology Unit, Department of Woman and Child Health, University of Padova, Padova, Italy; 3 Diabetology Unit, Department of Women’s and Children’s Health, University of Padova, Padova, Italy

**Keywords:** Limited joint mobility, Cheiroarthropathy, Sclerodactyly, Nailfold capillaroscopy, Microangiopathy, Dipeptidyl peptidase-4

## Abstract

**Background:**

Limited joint mobility (LJM), previously known as cheiroarthropathy, refers to the presence of reduced extension at the finger joints in people with diabetes and may be associated with scleroderma-like syndromes such as diabetic sclerodactyly.

While scleroderma-like syndromes and LJM have been observed in patients with long-term diabetes and associated complications, the coexistence of diabetes with Juvenile systemic sclerosis (jSSc) is rarely described.

**Case presentation:**

We describe the case of a 14-year-old boy with long-lasting type 1 diabetes (T1D) and suspected LJM associated with Raynaud phenomenon, sclerodactyly and tapering of the fingertips. A comprehensive work-up showed positive autoantibodies (ANA, anti-Ro-52, anti-Mi-2b), abnormal nailfold capillaroscopy with a scleroderma pattern, interstitial lung disease and cardiac involvement. The overall clinical picture was consistent with the diagnosis of jSSc.

**Conclusions:**

LJM can be the initial sign of underlying systemic sclerosis. Nailfold capillaroscopy may help differentiate jSSc from classical LJM in pediatric patients with T1D and finger contractures or skin induration of no clear origin.

This case report provides a starting point for a novel hypothesis regarding the pathogenesis of jSSc. The association between T1D and jSSc may be more than a coincidence and could suggest a relationship between glucose metabolism, fibrosis and microangiopathy.

## Background

Limited joint mobility (LJM), previously known as cheiroarthropathy, refers to the flexion contractures the develop in the finger joints of patients with diabetes [1]. The prevalence of LJM in adults with diabetes mellitus ranges from 8 to 58%, whereas in children and adolescents with type 1 diabetes (T1D), it is significantly lower (7%) [1].

Diabetic sclerodactyly associated with LJM has been described in about one-third of children, and especially in those with moderate to severe disease [2]. These skin changes are more common below the age of 15 with a prevalence ranging between 8 and 50% [3]. Conversely, the coexistence of T1D with juvenile systemic sclerosis (jSSc) is anecdotal [4–6].

We describe the case of a 14-year-old boy with T1D since age 4, who had slowly developed jSSc. We aim to outline an approach to differentiating between jSSc and LJM in pediatric patients with chronic T1DM. Moreover, we propose a novel hypothesis on the pathogenesis of jSSc, which suggests that the association between T1D and jSSc may involve a close relationship between glucose metabolism, fibrosis, and microangiopathy.

## Case presentation

**Fig. 1:**
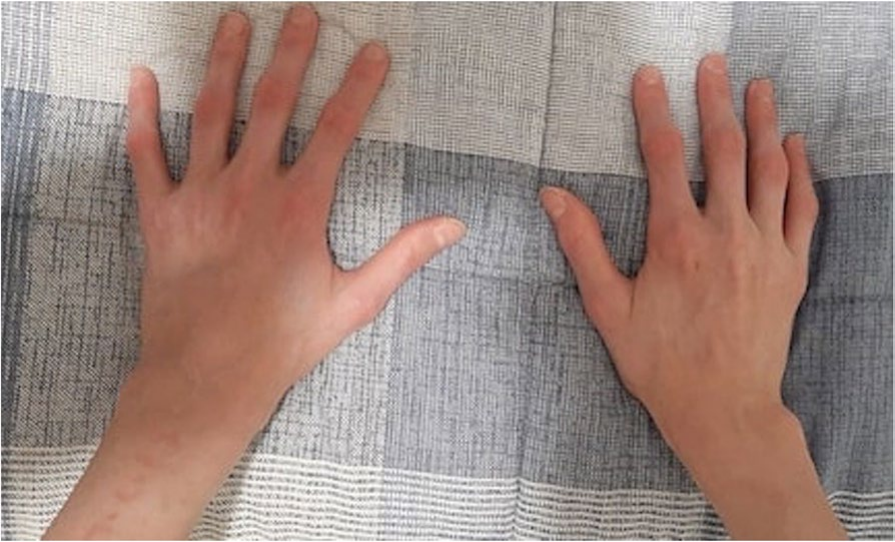
Photo of patient’s hands showing skin changes and contractures

**Fig. 2:**
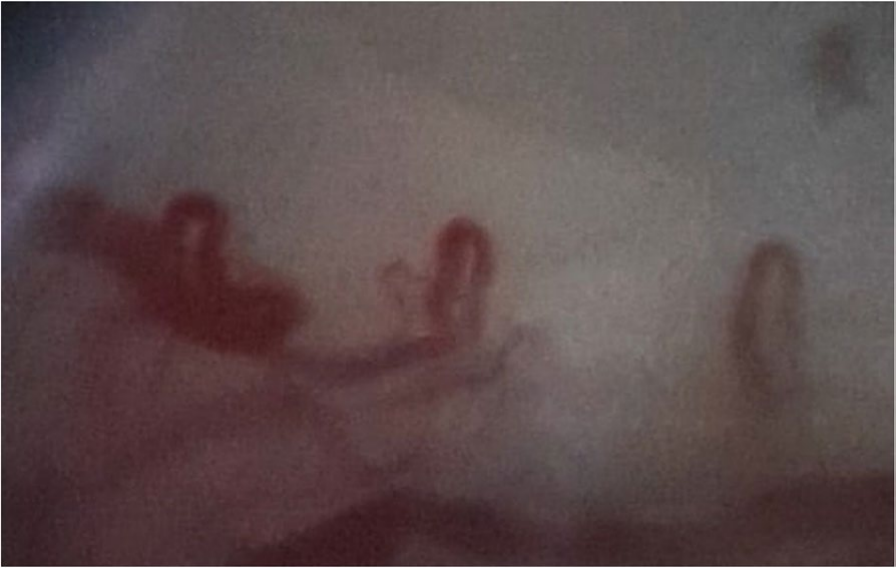
Nailfold capillaroscopy. Disorganization of the microvascular array, numerous megacapillaries, branching capillaries, microhemorrhages, and avascular area

A 14-year-old Caucasian boy was referred to our Pediatric Rheumatology Unit for finger joint contractures. Since the age of 4, he had been treated for T1D without complication and with fair glycemic control (hemoglobin A1c 7,5%). At the age of twelve, he presented with a first episode of puffy hands and Raynaud phenomenon (RP). Subsequently, he developed mild pain and reduced range of motion (ROM) at all the finger joints, along with tight skin on the dorsum of the fingers (Fig. 1). Physical examination confirmed the presence of sclerodactyly, periungual erythema, pachydactyly and tapered fingertips at the 1st and 2nd digits bilaterally. Neither digital ulcers, pitting scars nor signs of muscle weakness had been detected. He also had contractures of the metacarpophalangeal and interphalangeal joints (ranging between 40° and 45°), elbows and wrists, and a trigger finger of 1st and 5th fingers bilaterally. The “prayer sign” and “tabletop test” were positive. Clinically, there was no respiratory or cardiovascular involvement. Laboratory tests included normal values of blood cell count, erythrocyte sedimentation rate, C-reactive protein, muscle enzymes, serum pro-BNP, and urinalysis. Autoantibody profile showed high antinuclear antibodies (ANA) (1:640 with fine speckled pattern) with extractable nuclear antigen antibodies positive for anti-Mi-2b and anti-Ro52. Other scleroderma-specific autoantibodies were all negative including anti-Scl70 and anti-centromere, as well as anti-U1RNP. Ultrasound showed thickening of the flexor tendon sheaths, especially at the 3rd and 4th fingers bilaterally. Standard x-ray and magnetic resonance imaging (MRI) of the hands were unremarkable. Nail fold capillaroscopy revealed disorganization of the microvasculature, megacapillaries, branching capillaries, microbleeding, and avascular areas compatible with an “active scleroderma pattern” (Fig. 2). Cold challenged infrared thermography showed at 10 minutes, an abnormal temperature gradient (deltaT) > 1 °C in all fingers form metacarpophalangeal to distal interphalangeal joints [7]. Pulmonary function tests revealed a restrictive pattern with mild reduction of the diffusing capacity of the lungs for carbon monoxide (DLCO) (DLCO 72%, TLCO 73%, KCO 74%). Chest high-resolution computed tomography (HRCT) showed a bilateral interstitial lung thickening with perihilar distribution and a fibrotic area in the apex. Echocardiogram revealed a mild reduction (− 17.5%) of the global longitudinal strain of the left ventricle with no signs of pulmonary hypertension. Cardiac MRI ruled out myocardial fibrosis.

According to the current criteria for systemic sclerosis (SSc), our patient fulfilled 6 minor criteria of the pediatric EULAR/PRINTO/PReS classification (sclerodactyly, Raynaud phenomenon, nail fold capillary abnormalities, decreased DLCO, pulmonary fibrosis (HRCT/radiography), antinuclear antibodies positivity) and scored 11 on the recent SSc Classification [8, 9]. A diagnosis of jSSc was made and treatment with mofetil mycophenolate at 600 mg/m [2]/day was started with a significant improvement in DLCO (84%) and in the ROM of all fingers with only a residual 10° metacarpophalangeal flexion contracture at 6-month follow-up.

## Discussion and conclusions

LJM in diabetes most often develops as a consequence of long-lasting disease [10]. However, the correlation between good glycemic control and limited joint mobility remains controversial [11, 12]. Aside from dry skin, diabetic sclerodactyly is the most common skin manifestation of T1D. There is a correlation between T1D disease duration and the development of diabetic sclerodactyly as well [13]. Although the etiopathogenesis of LJM and associated scleroderma-like conditions have not been established, nonenzymatic glycation of collagen, diabetic neuropathy, and microangiopathy of dermal and subcutaneous vessels have been suggested as contributors [14, 15]. Altogether these factors result in skin hardening and deposition of abnormal collagen in the connective tissue around the joints [16]. Even if scleroderma-like syndromes and LJM in diabetes are observed in approximately 7% of children and adolescents, the coexistence of T1D with jSSc is anecdotal [4, 5].

In our case, this patient has clear features of jSSc and to date, only three other case reports have described a similar association between SSc and T1D (Table 1) [4–6]. The first report (patient 1) describes a patient with juvenile-onset T1D, who at 42 years of age developed RP, diffuse skin hardening and sclerodactyly. He presented with a typical scleroderma capillaroscopy pattern, antinuclear antibodies, myocardial, musculoskeletal, renal and gastrointestinal involvement [6].

**Table 1:** Present case compared to case reports reporting the coexistence of LJM and SSc or jSSc [4–6]

Patient no.	Author, year (Ref. no.)	Gender	Age at SSc diagnosis (years)	Diabetes duration at SSc diagnosis (years)	Diabetes Associated Conditions	Skin findings	Digital ulcers	RP	Internal Organ involvement	Auto antibodies	SSc-associated autoantibodies	Capillaroscopy Scleroderma pattern	ACR/EULAR 2013 Classification Criteria for SSc (score ≥ 9)
**1**	Wielosz E et al. 2017 [6]	M	42	29	Retinopathy Nephropathy Neuropathy Diabetic foot	Sclerodactyly skin induration	No	Present	Esophageal Renal Cardiac MSK	ANA	Negative	Active	18
**2**	Zeglaoui H et al. 2010 [5]	F	15	9	Neuropathy Celiac disease SLE	Sclerodactyly telangiectasia	Yes	Present	Esophageal MSK	ANA dsDNA RF, TPO, AGA, AEA, ARA, ATG	Anti-PM/Scl	N/A	11
**3**	Polak M et al. 1996 [4]	M	14	9	Exocrine pancreatic insufficiency	Skin induration, Sclerodactyly	No	Present	Esophageal Respiratory	Negative	Negative	Unspecific	16
**4**	Present case	M	14	11	None	Sclerodactyly	No	Present	Respiratory Cardiac MSK	ANA	Anti-Ro-52	Active	11

*RP* Raynaud phenomenon, *MSK* Musculoskeletal, *ANA* Antinuclear antibodies, *anti-dsDNA *anti-double-stranded DNA antibodies, *RF* Rheumatoid factor, *TPO* anti-thyroid peroxidase antibodies, *AGA* IgA and IgG antigliadin antibodies, *AEA* IgA antiendomysial antibodies, *ARA* IgA antireticulin antibodies, *AtTG* IgA anti-tissue transglutaminase antibodies

The second (patient 2) is a 15-year-old girl with T1D who developed sclerodactyly, telangiectasia, RP, bilateral digital ulcerations, arthritis and dysphagia. Several autoantibodies (including ANA and anti-PM/Scl) were positive [5]. The third patient (patient 3) is a 14-year-old boy with T1D and exocrine pancreatic insufficiency. The diagnosis of jSSc was made based on the appearance of skin induration, sclerodactyly, RP, esophageal and pulmonary involvement [4]. The case we describe has some clinical and laboratory features in common with the previously reported cases, such as sclerodactyly and RP. Both patient 1 and the patient in this case have an “active scleroderma” capillaroscopy pattern which strengthened the diagnosis of SSc. Whereas all the other described patients developed esophageal involvement in their disease course, our patient had cardiac and pulmonary manifestations -reported only in patient 1 and 3 respectively. Musculoskeletal symptoms were found in all but one patient (patient 3). All patients except patient 3 had a positive ANA. However, the SSc-associated autoantibodies were different between all patients.

The major diagnostic dilemma in our case was in differentiating diabetic skin hardening with LJM from the indurative skin changes typical of SSc. A few features specific for SSc such as RP, finger tapering, positive autoantibodies, interstitial lung disease, and cardiac involvement, allowed differentiation between the two conditions [2, 17]. The test that most facilitated the differentiation of jSSc from a scleroderma-like syndrome was capillaroscopy, which showed an “active scleroderma pattern” in our patient as well as in patient 1. Some capillaroscopy changes can also be observed in advanced diabetes, but they are characterized by a completely different pattern from SSc with reduced blood flow in capillaries and isolated homogeneous engorgement of venular limbs [18]. Infrared thermography with cold challenge can also help in the differential diagnosis [7]. The test can be abnormal in both SSc and diabetes, but due to completely different pathophysiology. In diabetes, there is a decreased perfusion reserve that appears under conditions of stress, with a more homogeneous re-warming pattern than in SSc [18]. Unfortunately, none of the other above-mentioned reports reported the use of thermography. Another distinguishing feature is the detection of typical SSc-related or associated autoantibodies. Among the cases reported in Table 1, only patient 2 had SSc-associated antibodies, and patients 1 and 3 tested negative. Our case was anti-Ro-52 positive, which is considered a SSc-associated autoantibody. Myositis-specific antibodies, as anti-Mi-2b found in our patient, are typically thought to be rare in jSSc, although Leurs et al. have recently reported a prevalence of 8.0% in SSc [19].

As for pulmonary involvement, in long-lasting T1D with LJM, restrictive pulmonary disease can occur. Some theories regarding the cause of the restrictive pulmonary disease include: increased elastic recoil or decreased chest-wall compliance due to muscle weakness [15], or skin thickening of the chest wall [1]. However, unlike SSc, there is no reduction of DLCO [20].

There are two possible hypotheses that could explain the overlap between LJM and jSSc: the coexistence of two autoimmune diseases, or a common microvascular insult. Although diabetes associated with SSc is rare [4–6], both conditions are autoimmune diseases. It is commonly known that autoimmune diseases with organ specific antibodies like T1D, can be associated with other rheumatologic conditions characterized by non-organ specific antibodies like jSSc or JIA [21, 22]. It has been reported that T1D is more likely to occur in first-degree relatives of patients with SSc [21] and a positive family history for T1D has been also reported in patients with localized scleroderma [22].

When compared to patients without LJM, patients with LJM are at high risk for the development of microvascular disease in T1D such as nephropathy, retinopathy and neuropathy [10, 23, 24]. The pathogenesis of jSSc is not fully elucidated but studies show that the endothelial and microvascular system play an important role both in the fibrotic process and in other features of the disease [25]. Since both T1D and jSSc can present with significant microvascular involvement, the hypothesis of a common mechanism underlying the induction of fibrosis may be more plausible. A recent study showed that dipeptidyl peptidase-4 inhibitors, which are widely used for the treatment of type-2 diabetes, promote the regression of fibrosis in animal models [26]. Moreover, dipeptidyl peptidase-4 inhibitors, precisely targeting hyperglycemia, have shown to be effective also in decreasing the incidence of autoimmune diseases in diabetic patients [27]. Given the paucity of reports in this field, further studies are needed to prove these hypotheses.

In conclusion, this case report suggests that nailfold capillaroscopy may help in differentiating jSSc from classical LJM. In addition to illustrating the differential diagnosis of LJM in diabetes, this case indicates that the association between T1D and jSSc may not be just a coincidence but might suggest a possible relationship between glucose metabolism, fibrosis and microangiopathy. However, we only describe a small cohort that does not allow any conclusions to be drawn. Nevertheless, our findings might provide new insights in order to help clarify the pathogenesis of SSc itself and may raise important implications for future treatment strategies targeting the glucose metabolism pathway to downregulate fibrosis in jSSc.

## Data Availability

The data and materials described in the current report are available from the corresponding author on reasonable request.

## Abbreviations

LJM: Limited Joint Mobility
T1D: Type 1 Diabetes
SSc: Systemic sclerosis
jSSc: Juvenile systemic sclerosis
RP: Raynaud phenomenon
ANA: Antinuclear antibodies
MRI: Magnetic resonance imaging
DLCO: Diffusing capacity of the lungs for carbon monoxide
TLCO: Transfer factor of the lung for carbon monoxide
KCO: carbon monoxide transfer coefficient
HRCT: High-resolution computed tomography
